# Anatomical Specimens, Etc.

**Published:** 1868-02-01

**Authors:** 


***Anatomical Specimens, etc.***

Particular attention is suggested to the advertisement of Messrs. HOVEY & NICHOLS on our advertising sheet. Their establishment is literally a *palace of art*, and one of the established "lions" of the city. A long acquaintance with the senior partner, and the high reputation of Mr. N., enable us to recommend the firm as entitled to entire confidence, and every way worthy the patronage of the profession. No physician visiting the city should fail to call upon them; and whether he purchases any thing or not, at least gladden his eyes by looking at things of beauty, which are joys for ever.

